# Cognitive biases in response to visual body-related stimuli in eating disorders: study protocol for a systematic review and meta-analysis

**DOI:** 10.1186/s13643-015-0093-4

**Published:** 2015-07-30

**Authors:** Kathrin Schuck, Simone Munsch, Silvia Schneider

**Affiliations:** Faculty of Psychology, Mental Health Research and Treatment Center, University of Bochum, Massenbergstrasse 9-13, 44787 Bochum, Germany; Department of Psychology, Clinical Psychology and Psychotherapy, University of Fribourg, Rue P.A. de Faucigny 2, 1700 Fribourg, Switzerland

**Keywords:** Eating disorder, Cognitive bias, Attention, Information processing, Body image

## Abstract

**Background:**

Biased processing of body-related information may be linked to the development and maintenance of eating disorders (ED). The objective of this systematic review will be to examine the occurrence and the extent of cognitive biases in response to visual body-related stimuli in individuals with ED and individuals with an increased risk to develop ED.

**Methods:**

Studies will be identified by searching MEDLINE and PsycINFO. We will include observational and experimental studies that examine the association between cognitive biases (information processing biases) in response to visual body-related stimuli and eating-related pathology in clinical and non-clinical adult samples. In addition to database searches, citation tracking will be used. Two reviewers will first screen titles and abstracts independently and will then review full texts for eligibility. Data extraction will be done independently by two reviewers. Conflicts at all levels of screening and extraction will be resolved through discussion. Studies will be included if they 1) assess cognitive biases (i.e., attentional biases, memory biases, judgment biases, response biases, and interpretation biases) in response to visual body-related stimuli (i.e., pictures or photographs of a human body or a human body shape), 2) if they report associations between biases and eating-related pathology, 3) if study participants are at least 16 years or older, and 4) if no priming task was administered prior to the assessment. Descriptive data of studies will also be collected. Risk of bias assessment will be undertaken by two independent reviewers. Data will be analyzed using random-effects meta-analysis.

**Discussion:**

This systematic review will synthesize the evidence for cognitive bias in response to visual body-related stimuli in individuals with ED and individuals with an increased risk of developing an ED. The findings may help to better understand information processing in eating-related psychopathology.

**Systematic review registration:**

PROSPERO CRD42015019165

## Background

Cognitive bias refers to a well-established finding that individuals who suffer from certain clinical problems display biased information processing in response to events relevant to their condition. A large body of research has shown that, in various mental conditions, individuals selectively attend to, remember, and interpret events in ways that are congruent with their disorder [1, 2]. In eating disorders (ED), disturbed body images, preoccupation with weight and shape, and body dissatisfaction are core characteristics. Biased information processing related to body size, weight, and shape may influence the development and maintenance of eating disorders.

From a theoretical perspective, distorted schemata are assumed to underlie cognitive biases. Generally, schemata are stable cognitive structures such as core beliefs, underlying assumptions, and automatic thoughts, which provide a basis for screening, coding, and evaluating stimuli in the environment [3]. Individuals with ED are assumed to have developed distorted or maladaptive schemata (e.g., body-shape schema containing stereotyped, affectively loaded, and overvalued information with regard to weight and shape) [4]. Distorted schemata and inaccurate cognitive structures may maintain symptoms of mental disorders, because they determine what people notice, attend to, and remember of their experiences. Once formed, schemas are quite pervasive and maintained even in the face of contradictory evidence through processes of distorting, not noticing, or discounting contradictory information [3].

In ED, biased information processing related to body size and weight has been proposed to reinforce overconcern with weight and shape and disturbed body image experiences [5], thereby contributing to dietary restriction and restraint, body dissatisfaction, and negative affect [6], which has been associated with the onset and maintenance of disturbed eating behavior [7]. For example, Jansen and colleagues [8] showed that eating-disordered patients show a dysfunctional way of looking at their own bodies. When looking at their own body, eating-disordered patients attended more to their self-defined unattractive body parts than to their self-defined attractive body parts, whereas healthy controls attended more to their own attractive body parts than to their unattractive body parts. The pattern of results was reversed when participants were exposed to another body; eating-disordered patients attended relatively more to the other’s attractive body parts, whereas healthy controls attended relatively more to the other’s unattractive body parts, indicating an information processing bias, which might maintain ED-related pathology. Indeed, preliminary experimental data indicates that the induction of an attentional bias toward shape/weight-related information resulted in higher body dissatisfaction among female students after a body image task, indicating that an attentional bias may plays a causal role in the development of ED [9].

To date, a large body of evidence including several systematic reviews and meta-analyses has provided support for the existence of cognitive biases in people suffering from ED [5, 10–12]. The vast majority of research in this area has focused on cognitive biases in response to semantic stimuli (i.e., body- or food-related words). Studies examining cognitive biases in response to visual stimuli (i.e., pictures or photographs of a human body or a human body shape) are far less common. The presentation of pictures and photographs is of higher ecological validity and may therefore have a better generalizability to real-life situations, such as the effects of media portrayals of the ideal physique on young women. While there is robust evidence for cognitive biases in response to semantic disorder-relevant stimuli and pictorial food stimuli, far less is known regarding information processing of visual body-related stimuli in ED.

With regard to semantic stimuli, there is robust evidence for attentional biases in response to body- or food-related words. For example, a systematic review and meta-analysis evaluating Stroop performance (i.e., color-naming retardation in response to disorder-relevant semantic stimuli, indicating attentional interference or distraction by stimulus meaning) concluded that, in comparison to non-eating disordered women, women with ED display Stroop interference of medium effect size for food- and body-related words with negative valence (e.g., high-calorie food words, weight and shape words associated with large physique) compared to neutral words [11]. Also, there are indications of Stroop effects among individuals concerned about eating, shape, and weight [10, 12], indicating that cognitive inference may not be limited to clinical samples with ED. Single studies have also provided preliminary support for the existence of memory biases to eating-related words in women with ED and restrained eaters [13, 14]. Far less is known with regard to cognitive biases in response to visual stimuli. Up to this point, only one meta-analytic review has examined responses to pictorial food cues. This review provides evidence that individuals with ED show distortions in the processing of pictorial food stimuli, such as attentional biases, cue reactivity, and higher emotional involvement [15].

With regard to visual body-related stimuli (i.e., responses to pictures or photographs of a human body or a human body shape), the evidence for cognitive biases has not been systematically examined. Yet, information processing of body-related visual stimuli may be particularly relevant in understanding the key characteristics of ED, such as preoccupation with weight and shape, body image disturbances, and body dissatisfaction. Biased information processing related to body size, weight and shape may be important in understanding the onset as well as the maintenance of eating-related pathology, which could be important in improving prevention and treatment efforts. The aims of this systematic review are twofold. First, we aim to synthesize and summarize the evidence for cognitive biases in response to visual body-related stimuli in individuals with ED. Second, we aim to examine whether these biases are specific to clinical samples with ED or whether non-clinical samples displaying symptoms of eating-related pathology (e.g., overconcern with weight and shape, high levels of body dissatisfaction, high drive for thinness, restrained eating) would also show cognitive biases in response to visual body-related stimuli compared to individuals with no eating-related pathology.

## Methods

### Aims

The proposed systematic review aims to synthesize the evidence for cognitive biases in response to visual body-related stimuli in individuals with ED. In addition, we will examine the evidence for a graded association between cognitive biases and symptoms of eating-related pathology (e.g., overconcern with weight and shape, high levels of body dissatisfaction, high drive for thinness, restrained eating, thin-ideal internalization), to investigate whether individuals with an increased risk for developing an ED may also display cognitive biases in response to visual body-related stimuli. The proposed review will be reported in accordance with the reporting guidelines provided in the Preferred Reporting Items for Systematic reviews and Meta-analyses (PRISMA) statement [16]. The proposed review is registered in the PROSPERO database (CRD42015019165).

### Search strategy

We will search the databases PubMed/MEDLINE and PsycINFO for relevant articles. No restrictions with regard to language or publication period will be imposed during the searches. A draft search strategy is included in the Appendix. In addition to the database search, we will hand-search the reference lists from identified relevant articles (“backward citation tracking”). In addition, the Social Science Citation Index will be searched for all articles which cite any of the identified relevant articles (“forward citation tracking”).

### Study eligibility criteria

#### Inclusion criteria

The systematic review will consider observational studies (cross-sectional and prospective) and experimental studies. Only full-text journal articles (no conference abstracts, doctoral dissertations, or book chapters) published in English language will be eligible. If full-text articles are unavailable, attempts to obtain full-text articles from the authors will be made. To be included in the systematic review, studies have to meet the following five inclusion criteria: 1) The degree of eating-related pathology among participants has to be assessed; clinical diagnosis of ED, symptoms of ED, dietary restraint, body dissatisfaction, weight- and shape concerns, drive for thinness, and thin-ideal internalization are considered eating-related pathology; definition of eating-related pathology can be based on a clinical interview, a medical record, or self-report; normal controls have to be screened for symptoms of ED either using a clinical interview or by self-report measures assessing eating-related pathology; individuals with a clinical diagnosis of ED or with elevated scores on self-report measures indicative of subclinical or clinical levels of an ED will need to be excluded from the normal control group; 2) studies will only be included if they assess responses that are considered cognitive biases (information processing biases) including attentional biases, memory biases, judgment biases, response biases, and interpretation biases and if they employ visual body-related stimuli (i.e., pictures or photographs of a human body or a human body shape); 3) studies will only be included if they examine the association between eating-related pathology and cognitive biases; this association can be examined using either between-group comparison (e.g., analysis of variance between samples to obtain a *d*-statistic) or within-group analyses (e.g., correlational analyses between eating-related pathology and cognitive bias measures to obtain an *r*-statistic); 4) no priming task should have been administered prior to the assessment of cognitive biases; and 5) studies will only be included if study participants are at least 16 years or older.

#### Exclusion criteria

Studies will be excluded if 1) they do not assess eating-related pathology of the sample, 2) if they do not investigate an association between cognitive biases and eating-related pathology, 3) if they do not exclude individuals with a clinical diagnosis of ED or individuals with high levels of eating-related pathology (indicative of clinical or subclinical levels of ED) from normal controls, 4) if study participants are younger than 16 years (child or adolescent samples), and 5) if they do not examine cognitive biases in response to visual body-related stimuli. Pictures or photographs of human faces (without body) are not considered to be body-related stimuli.

### Data collection

Two reviewers will screen titles and abstracts independently, excluding those that are irrelevant. All studies potentially eligible for inclusion will be retained for full-text examination. Two reviewers will examine full texts for eligibility independently. Reasons for exclusion will be recorded. The same reviewers will also be responsible for extraction of the data. Data will be extracted independently using a pre-defined data extraction forms that will be piloted on a small number of studies and revised if necessary. Disagreements between reviewers at any of the above steps will be resolved through discussion. A PRISMA flow chart will display the study selection process and reasons for exclusion.

Extracted data will include information pertaining to study identification (first author, year of publication, country where data collection took place), study characteristics (study design, sample size, setting), sample characteristics (sample definition, type of eating-related pathology, diagnostic instrument used), participant characteristics (age, gender, body mass index, educational level), task characteristics (employed task, duration of stimulus presentation, frequency of stimulus presentation), stimuli characteristics (type of stimuli, number of stimuli), the type of outcome (type of cognitive bias), and adjustment for covariates. Any discrepancies between reviewers will be discussed until consensus is achieved. In case of missing data or unclear information, we will contact the first authors of a publication. The following outcomes will be considered cognitive biases: attentional biases (e.g., eye-tracking task, dot-probe task), memory biases (e.g., recall task, recognition task, implicit association task), judgment biases (e.g., estimation tasks), response biases (e.g., approach-avoidance task), and interpretation biases (e.g., sentence-completion task). Cognitive biases will need to be assessed in response to visual body-related stimuli (i.e., pictures or photographs of a human body or a human body shape).

The following parameters will be extracted: in between-group designs, means, standard deviations, and sample size will be collected for each group to calculate effect size *d* (Kock, [17]). As we expect heterogeneity in outcome measures, we will use the standardized mean difference score, which transforms all effect sizes to a common metric and enables the inclusion of different outcome measures (Borenstein et al., [18]). In within-group designs, we will collect effect size estimates (e.g., *r*, *beta*, *F*, *t*, or *p* values) to calculate effects size *r* (Kock, [17]). Effect size *r* will be used as the effect size. As both measurements (*d* and *r*) can be arbitrarily converted to one another, we will convert all effect sizes to the *d*-index when most of the included studies report an independent variable that is dichotomous, whereas we will convert all effect sizes to the *r*-index when most of the included studies report an independent variable that is continuous.

### Quality assessment (risk of bias assessment)

Quality assessments (risk of bias assessments) for each study included will be conducted independently by two reviewers. Again, any discrepancies between reviewers will be resolved through discussion. The Newcastle Ottawa Scale [19] will be used to assess the quality of the included studies based on selection of participants, comparability of participants, and assessment of outcome. The a priori chosen score of 7 will be used to distinguish high from lower quality studies [20].

### Data synthesis

We will provide a detailed description of the results in both tables and text for all included studies. Studies will be described to provide insight into descriptive characteristics of the study population (e.g., age, gender, body mass index, eating pathology), the task characteristics, the stimulus material, the type of outcome, and the comparisons made. Additional information (year of publication, country in which data collection took place) will also be provided. The quality of evidence will be described (high, low, unclear).

### Meta-analysis

We plan to conduct meta-analyses for each type of cognitive bias (attentional biases, memory biases, judgment biases, response biases, and interpretation biases). A minimum number of five studies will be required to conduct a meta-analysis. We will favor a random-effects meta-analysis, as we expect differences in the methodology of studies. We will calculate effect sizes for each study using mean difference scores and standard deviations, if available. If means and standard deviations are not available, effect sizes will be calculated using effect size estimates (*F* value, *t* value, *p* value, correlation coefficients, standardized regression coefficients). Results from separate studies will be pooled into a weighted average and reported together with the 95 % confidence interval (CI). Results will be displayed in a forest plot. Heterogeneity will be evaluated using the *Q*-Statistic and the |^2^ statistic. According to the Cochrane Handbook for Systematic Reviews [21], |^2^ of 0 to 60 % can be regarded as not important to moderate, while |^2^ > 60 % indicates substantial heterogeneity.

### Subgroup analysis

We will enter data such that that the following subgroup analyses can be conducted: 1) clinical diagnosis of ED (clinical diagnosis of ED based on clinical interview or medical record vs. no clinical diagnosis of ED), 2) increased symptoms of eating-related pathology without a clinical diagnosis of ED (high symptomatic individuals based on self-report or clinical interview vs. healthy controls), and, if possible, 3) diagnostic subgroup of clinical ED diagnosis (Anorexia Nervosa vs. Bulimia Nervosa vs. Binge Eating Disorder vs. another specified or unspecified ED).

### Risk of publication bias

For all data combined, we will investigate systematic differences between reported and unreported findings by the test of Egger et al. [22] and visual inspection of funnel plots.

## Discussion

The objective of the systematic review will be to comprehensively and systematically synthesize the evidence for cognitive biases in response to visual body-related stimuli in individuals with ED and individuals with an increased risk to develop ED. A large body of evidence indicates the existence of attentional biases in clinical and non-clinical samples. Up to this point, existing reviews have focused solely on information processing of semantic stimuli and pictorial food stimuli. A systematic review examining information processing of visual body-related stimuli has not been conducted. Examining cognitive biases in response to pictures and photographs is of high ecological validity, and findings may generalize well to information processing in real-life situations (e.g., media exposure to thin-ideal). Examining responses to body-related stimuli is particularly relevant in understanding the more fundamental processes associated with chore features of ED, such as preoccupation with weight and shape, body image distortions, and body dissatisfaction, which might be amenable to change by specific prevention or treatment methods.

There are several strengths and limitations of our planned methods. In terms of strength, we will comprehensively and systematically review all available evidence in a relatively novel area of research. In terms of limitations, it is likely that the systematic review may be limited by publication bias of significant findings, given the novelty of the field. We will focus on studies including adult samples (study participants of 16 years or older) and will not be able to examine the developmental trajectory of cognitive biases, which would be preferable. Finally, we anticipate identifying studies with different methodologies and small sample sizes, which may increase statistical heterogeneity and limit conclusions.

We hope that our findings may help to better understand the more fundamental processes in ED such as information processing processes. A better understanding of the processes involved in the etiology and maintenance of ED is important for further advancements in the treatment of ED. In addition, the findings may guide future research into the mechanism underpinning chore symptoms of ED such as preoccupation with weight and shape, body image distortions, and body dissatisfaction.

The proposed review may have several theoretical and clinical implications. First, a systematic review of cognitive biases in ED may help to further build and strengthen explanatory models regarding the development and maintenance of ED. In addition, knowledge regarding the specific types of cognitive biases in individuals with ED might help to understand processes that can be targeted by psychotherapeutic interventions. Initial evidence suggests that implicit training tasks and exposure tasks , which may target the more fundamental processes associated with eating-related pathology such as attention, memory, perception, and automatic response tendencies, can reduce symptoms associated with ED, maladaptive behaviors, and symptom severity in various clinical disorders including ED [1, 23–25]. Understanding cognitive distortions in individuals with ED may guide the development of novel treatment approaches such as cognitive bias modification trainings to reduce symptom severity and increase treatment effectiveness in ED.

## Appendix

Key terms for PubMed/MEDLINE search:

1. Eating-related pathology terms: (“Eating disorder” OR “anorexia” OR “bulimia” OR “Binge eating” OR “Restricted eating” OR “Restrained eating” OR “Restrained eater” OR “Dietary restraint” OR “Body dissatisfaction” OR “Weight concern” OR “Shape concern”

2. Cognitive bias terms: (“Cognitive bias” OR “Cognitive distortion” OR “Perceptual Distortion” OR “Attentional bias” OR “Information processing bias” OR “Memory bias” OR “Judgement bias” OR “Interpretation bias” OR “Interpretative bias” OR “Response bias”)

3. Shape/body terms: (Shape OR body)

## Abbreviations

CI: confidence interval
ED: eating disorder
PRISMA: preferred reporting items for systematic reviews and meta-analyses
